# Effectiveness of kinesiology taping on the functions of upper limbs in patients with stroke: a meta-analysis of randomized trial

**DOI:** 10.1007/s10072-022-06010-1

**Published:** 2022-03-26

**Authors:** Yuxin Wang, Xiguang Li, Cuiyun Sun, Rong Xu

**Affiliations:** Department of Rehabilitation Medicine, Nanjing Drum Tower Hospital, The Affiliated Hospital of Nanjing University Medical School, Nanjing, China

**Keywords:** Kinesiology taping, Stroke, Systematic review, Meta-analysis, Upper limbs

## Abstract

**Background:**

Kinesiology tape (KT), a water-resistant and elastic tape which is well known measure for preventing musculoskeletal injuries, has recently gained popularity in neurological rehabilitation. This is a systematic and meta-analysis study, useful both to evaluate the efficacy of kinesiology taping on the functions of upper limbs in patients with stroke and to collect the main outcomes evaluated in the analyzed studies.

**Methods:**

A comprehensive literature search of electronic databases including Medline, Web of science, Embase, Cochrane Central Register of Controlled Trials, Physiotherapy Evidence Database (PEDro), WANFANG, and the China National Knowledge Infrastructure (CNKI). Additional articles were obtained by scanning reference lists of included studies and previous reviews. Keywords were “kinesiology taping,” “kinesio,” “kinesio taping,” “tape” and “stroke,” “hemiplegia,” “hemiplegic paralysis,” “apoplexy,” “hemiparesis,” “upper extremity,” “upper limb.” All the RCTs were included. Quality assessment was performed using Cochrane criteria. Upper extremity function and pain intensity was pooled as the primary outcome, and shoulder subluxation, muscle spasticity, general disability, PROM of abduction, and adverse effects as secondary outcomes.

**Results:**

Twelve articles were included. Pooled data provided evidence that there was significance between kinesiology taping groups and control groups in pain intensity (standardized mean difference − 0.79, 95% CI − 1.39 to − 0.19), shoulder subluxation (standardized mean difference − 0.50, 95%CI − 0.80 to − 0.20), general disability (standardized mean difference 0.35, 95%CI 0.10 to 0.59), upper extremity function (standardized mean difference 0.61, 95%CI 0.18 to 1.04), and the PROM of flexion (standardized mean difference 0.63, 95%CI 0.28 to 0.98).

**Conclusion:**

Current evidence suggested that kinesiology taping could be recommended to improve upper limb function in patients with stroke in pain intensity, shoulder subluxation, general disability, upper extremity function, and the PROM of flexion.

**Ethics and dissemination:**

Ethical approval requirements are not necessary for this review. This systematic review and meta-analysis will be disseminated online and on paper to help guide the clinical practice better.

**PROSPERO registration number:**

CRD42020179762.

## Introduction

Upper limb function after stroke is the main cause of long-term disability, so rehabilitation research is a top priority. Although nerve reorganization occurs soon after stroke, the natural rehabilitation of upper limb function recovery is usually limited. In order to overcome these limitations, new strategies are needed to strengthen nerve regeneration and restore brain structure and function. In addition, upper limb paresis is observed in 87% of stroke survivors [1]. Furthermore, impaired use of the upper limb persists in about 60% of the patients 6 months post-stroke [2]. Impairment of upper limb is a cause of muscle weakness, loss of multi-joint movements coordination, and changes in muscle tone and sensation [3], which is strongly correlated to dependency in activities of daily living (ADL) and participation restrictions [4]. Although previous studies reported that initial degree of severity of paresis, functional and structural changes, and genetic factors may influence recovery of upper limb function, predicting such recovery in stroke patients is difficult [5–7].

Clinicians now have more evidence to support the motion exercises, motor imagery, mirror therapy, and non-invasive brain stimulation, such as transcranial magnetic stimulation and transcranial direct current stimulation (tDCS) promote upper limb recovery. In addition, pain is one of the main outcomes that influence the choice of the type of treatment [8].

Kinesiology tape (KT) is an elastic, waterproof, and breathable tape and Kenzo Kase invented this tape in the 1970s. It can be stretched to 120–140% of its original length and can be recoiled after use [9]. KT is a widely used treatment in both clinical and sports fields. KT is similar to the elasticity of human skin, so it allows more movement and feels more comfortable. In recent years, KT has become increasingly popular in rehabilitation for hemiplegic patients. The recent systematic review which found a significant effect on taping for reduction of pain and subluxation in stroke patients was in line with the positive effect of KT and the study focused on various of tapes including therapeutic strapping, inelastic tape, California tri pull taping method and KT [10]. With the development of KT, it is increasingly used in upper limb rehabilitation. Some researchers proposed that KT could offer intensification of sensory input from the paretic upper limb to enhance motor performance and alleviate sensory impairment [11, 12]. Simoneau and Callagan found the positive effect of KT on proprioception [13, 14]. Owing to elastic stimulation of KT, it could allow for facilitation of muscle activation [15].

Research has shown that KT can promote functional use of the upper and lower extremity, in further to improve balance ability [16]. Recent meta-analyses of randomized controlled trials compared KT versus conventional rehabilitation for treating balance impairment after stroke with 22 studies included. The systematic reviews have demonstrated KT was more effective than conventional rehabilitation for balance function. They also pointed out that KT can improve lower limb function, and walking function in stroke patients [17]. A 2015 systematic review reported that there is insufficient evidence for adhesive taping post-stroke in improving outcomes, including pain intensity, range of motion, muscle tone, strength, activity, and participation [18]. As we all know, upper limb ability plays a very important role in the patient’s balance ability. This review focused on evaluate the effect of KT on upper limb function. In general, the efficacy of KT for function of stroke patients remains uncertain [19–23].

This is a systemic and meta-analysis study. The purpose of this review was to evaluate the effect of KT on upper limbs function outcomes in stroke patients. To evaluate the efficacy of KT for upper extremity function, pain intensity, shoulder subluxation, muscle spasticity, general disability, and the passive range of motion (PROM) of flexion and abduction in patients with stroke.

The systematic review was registered on PROSPERO (ID: CRD42020179762). All the PRISMA standards and recommendations for systematic review development were followed [24].

## Method

### Data sources

We searched Medline (via PubMed), Web of Science, Embase, the Cochrane database of Controlled Trials, PEDro, WANFANG, and CNKI up to July 30, 2021. Additional articles were obtained by scanning reference lists of included studies and previous reviews.

### Study selection

Keywords were (1) “kinesiology taping,” “kinesio,” “kinesio taping,” “tape” and (2) “stroke,” “hemiplegia,” “hemiplegic paralysis,” “apoplexy,” “hemiparesis,” “upper extremity,” “upper limb.” Studies were included if they met the following criteria: (1) In adults (18 years and older); (2) if they were RCTs conducted in patients with stroke comparing KT with conventional rehabilitation. (3) There was no restriction on follow-up and study size. (4) Studies needed to report 1 or more of the following outcomes: pain intensity, shoulder subluxation, muscle spasticity, general disability, upper extremity function, and the PROM of flexion and abduction. Studies were excluded if (1) trails using other forms of tape (e.g., inelastic tape). (2) Trails that did not provide abundant information to analyses treatment effects and we got no reply from the authors. (3) Trails that were non-randomized observational trials, cross-over design trails, case reports, clinical observations, and systematic reviews. This study conformed to all PRISMA guidelines and reported the required information accordingly (see Supplementary PRISMA checklist).

### Data extraction

Two investigators no involved in any of the selected studies independently screened each title and abstract to exclude duplicates and studies not meeting the inclusion criteria. Additionally, one of the reviewers extracted study and patient characteristics, intervention and comparator details, and outcome data from included studies using pre-specified data extraction tables. The second author check for accuracy, and disagreement was resolved by discussion. Using the criteria suggested in the Cochrane handbook, we assessed the bias of risk. If there were fewer than 10 studies included, publication bias was deemed non-estimable and not rated down [25].

### Statistical analysis

Quality assessment was undertaken using Cochrane criteria [26–28]. It is a two-part tool with seven specific domains: random sequence generation, allocation concealment, blinding of participants and personnel, blinding of outcome assessment, incomplete outcome data, selective reporting, and other bias [29]. Two reviewers conducted the quality assessment independently of each other and then crosschecked their findings. A Chi square test evaluated the statistical significance of heterogeneity. The *I*^2^ statistical significance. We defined an *I*^2^ > 50% as substantial heterogeneity. A value of 0% means there is no observed heterogeneity and lager percentages indicate more heterogeneity. To address treatment heterogeneity in the included studies, we tried to find an explanation for this heterogeneity by performing sensitivity analysis. Sensitivity analysis was conducted by removing individual studies one by one. Results of meta-analyses may overestimate the true population effect due to publication bias [30]. To reduce its potential impact, publication bias was determined by funnel plots and the Egger’s regression test [31]. Statistical significance was set at *P* < 0.05. Data were analyzed in Review Manager Version 5.3 software and Stata 15 software.

## Results

### Baseline characteristics

**Fig. 1:**
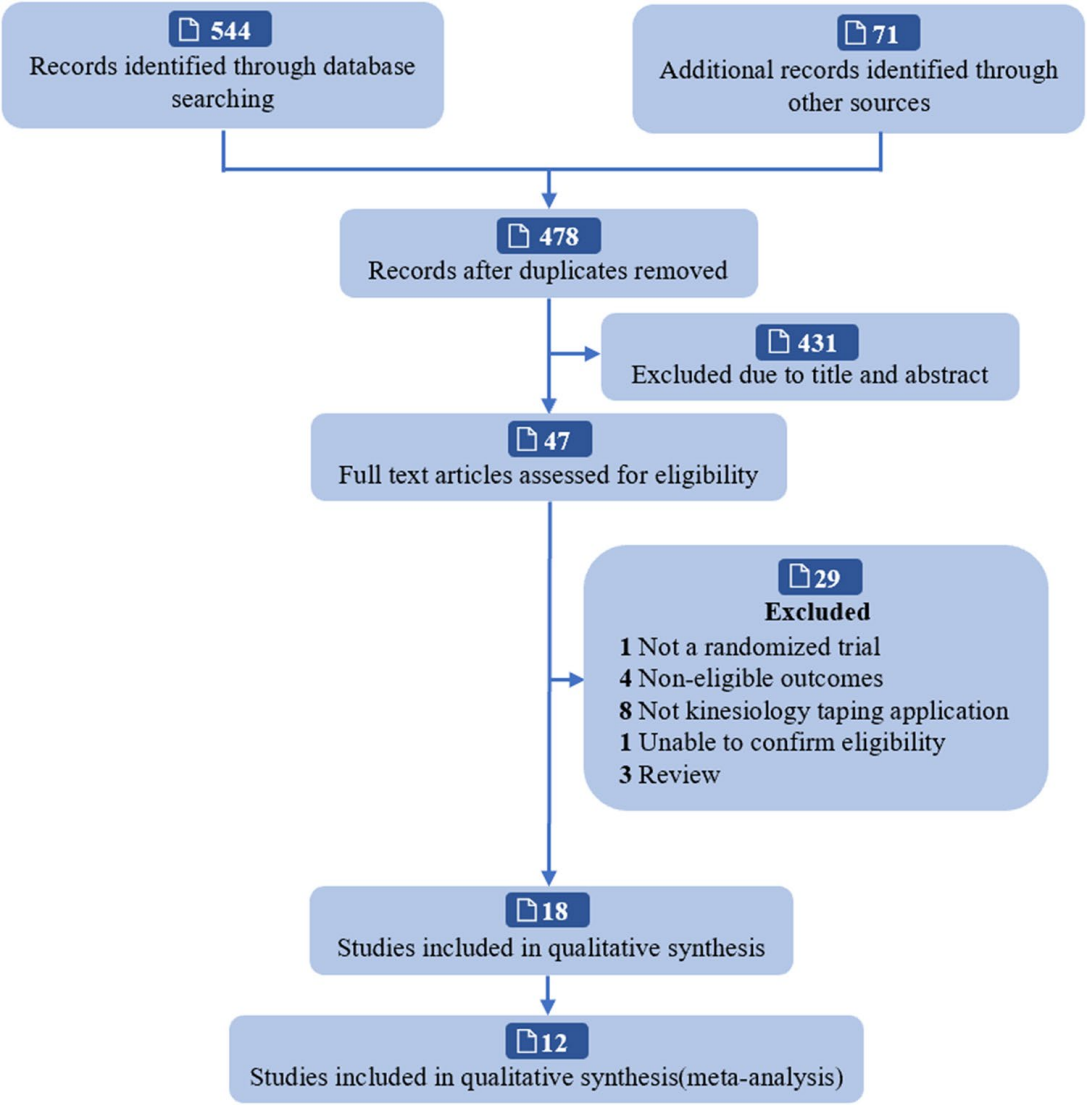
Flow diagram of the literature search and selection process

The study selection flow diagram is illustrated in Fig. 1. A total of 12 studies including 535 participants were included in the meta-analysis [21, 23, 32–40]. Sample size ranged from 14 to 120 participants. The average age ranged between 50.25 and 68.27 years old. In terms of type of KT, three trials used the NITTO medical adhesive tape, two trials used the Kinesio taping NFDA Production License 1,640,045, one trial used the Kinesio Tex gold tape, one trial used the 3NS kinesiology taping, one trial used the Kindmax Kinesio taping (Shanghai Kangmashi Sports Goods Co. LTD), one trial used the Kinesio taping (Nanjin Siruiqi Medical Supplies Co. LID), and three trials did not report. The intervention frequency varied between five to seven days per week and the follow-up period ranged between 3 to 28 weeks. Characteristic of included studies are summarized in Table 1.

**Table 1:** Characteristics of included studies in this systematic review and meta-analysis

First author/year	Sample size (*n*)	Mean age (SD)	Sex (M: F)	Intervention			Control	Follow-up period (weeks)
				Type of tape	Session length (hours)	Frequency (days/week)		
Chen 2018 [32]	84	52.95 (10.83)	50:34	Kinesio taping NFDA Production License 1,640,045	24–48	7	Conventional rehabilitation	4
Hochsprung 2017 [23]	14	62.00 (10.25)	11:3	Kinesio Tex Gold Tape, Kinesio, USA	72	6	Conventional rehabilitation	4
Huang 2016 [33]	44	61.34 (10.74)	30:14	NITTO Medical Adhesive Tape, Denko Corporation, Osaka, Japan	72	5	Conventional rehabilitation	3
Huang 2017 [34]	21	57.43 (13.90)	14:7	NITTO Medical Adhesive Tape, Denko Corporation, Osaka, Japan	72	5	Conventional rehabilitation	3
Huang 2019 [21]	31	50.58 (16.10)	24:7	NITTO Medical Adhesive Tape, Denko Corporation, Osaka, Japan	NR	7	Conventional rehabilitation	3
Kim 2015 [35]	30	68.27 (8.43)	13:17	3NS Kinesiology taping	NR	NR	Conventional rehabilitation	28
Li 2019 [36]	120	52.25 (8.22)	61:59	Kindmax Kinesio taping (Shanghai Kangmashi Sports Goods Co.,LTD)	24	6	Conventional rehabilitation	4
Shi 2018 [37]	56	65.19 (10.48)	36:20	NR	48	7	Conventional rehabilitation	6
Si 2019 [38]	50	67.76 (9.52)	28:22	Kinesio taping NFDA Production License 1,640,045	24–48	7	Arthrolysis	4
Yang 2016 [39]	26	54.65 (10.84)	15:11	NR	72	5	Conventional rehabilitation	8
Yang 2018 [37]	19	59.47 (2.85)	13:6	NR	10–12	NR	Conventional rehabilitation	4
Zhao 2017 [40]	40	67.45 (9.34)	24:16	Kinesio taping (Nanjin Siruiqi Medical Supplies Co.,LID)	48	7	Conventional rehabilitation	4

The SD for this study was inputted from another trial

*NR*, not reported

### Methodological quality and risk of bias

**Fig. 2:**
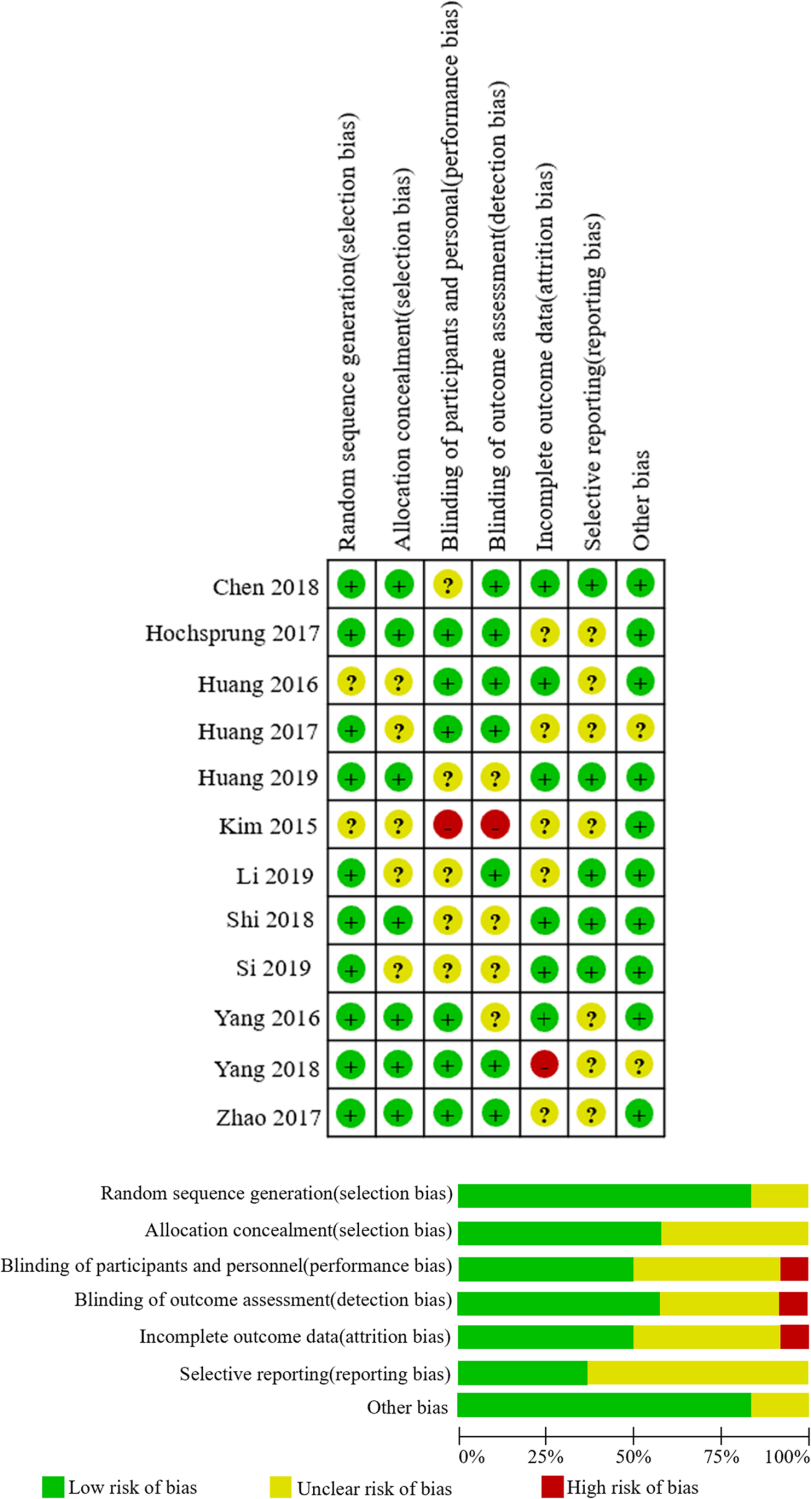
There were twelve randomized controlled studies included in our meta-analysis. The quality of studies was assessed with the Cochrane collaboration’s tool

Quality assessment was presented in Fig. 2. Ten studies reported whether there was random sequence generation (83.3%, 10/12) [21, 23, 32, 34, 36–41]. Seven studies reported whether there was appropriate allocation concealment (58.3%, 7/12) [21, 23, 32, 37, 39–41]. Only 41.7% (5/12) studies were double blind and 58.3% (7/12) had inadequate blinding. Although the information for assessing individual study quality was limited, the overall evidence should be considered of fairly high quality since most study had not significant missing data and only randomized trials were included. Additionally, none of the meta-analysis groups had greater than 10 studies and therefore neither Funnel plot or Egger test was completed.

## Outcomes

### Upper extremity function

**Fig. 3:**
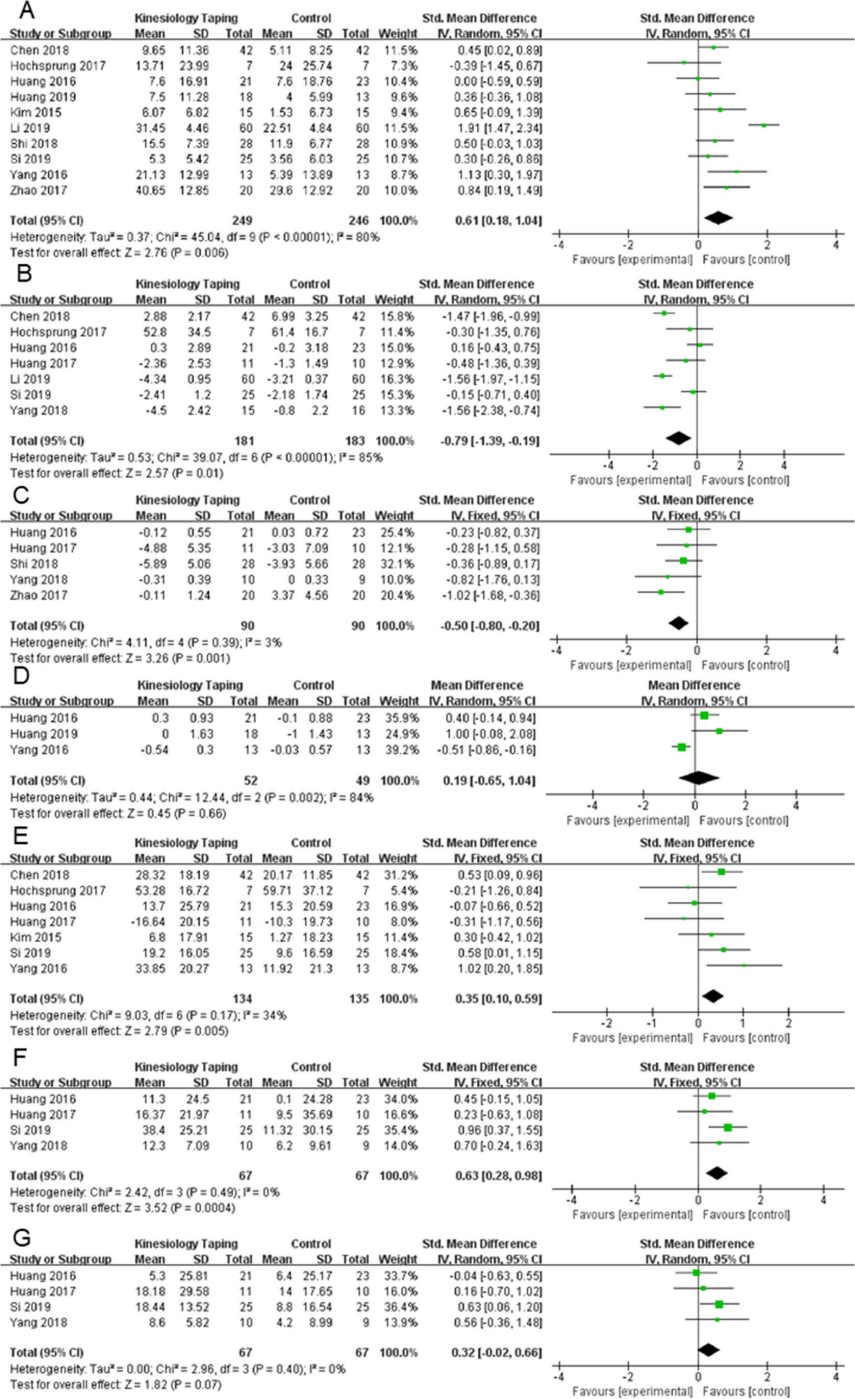
**A** Forest plot of strategies for efficacy of upper extremity function. **B** Forest plot of strategies for efficacy of pain intensity. **C** Forest plot of strategies for efficacy of shoulder subluxation. **D** Forest plot of strategies for efficacy of muscle spasticity. **E** Forest plot of strategies for efficacy of general disability. **F** Forest plot of strategies for efficacy of PROM of flexion. **G** Forest plot of strategies for efficacy of PROM of abduction

**Fig. 4:**
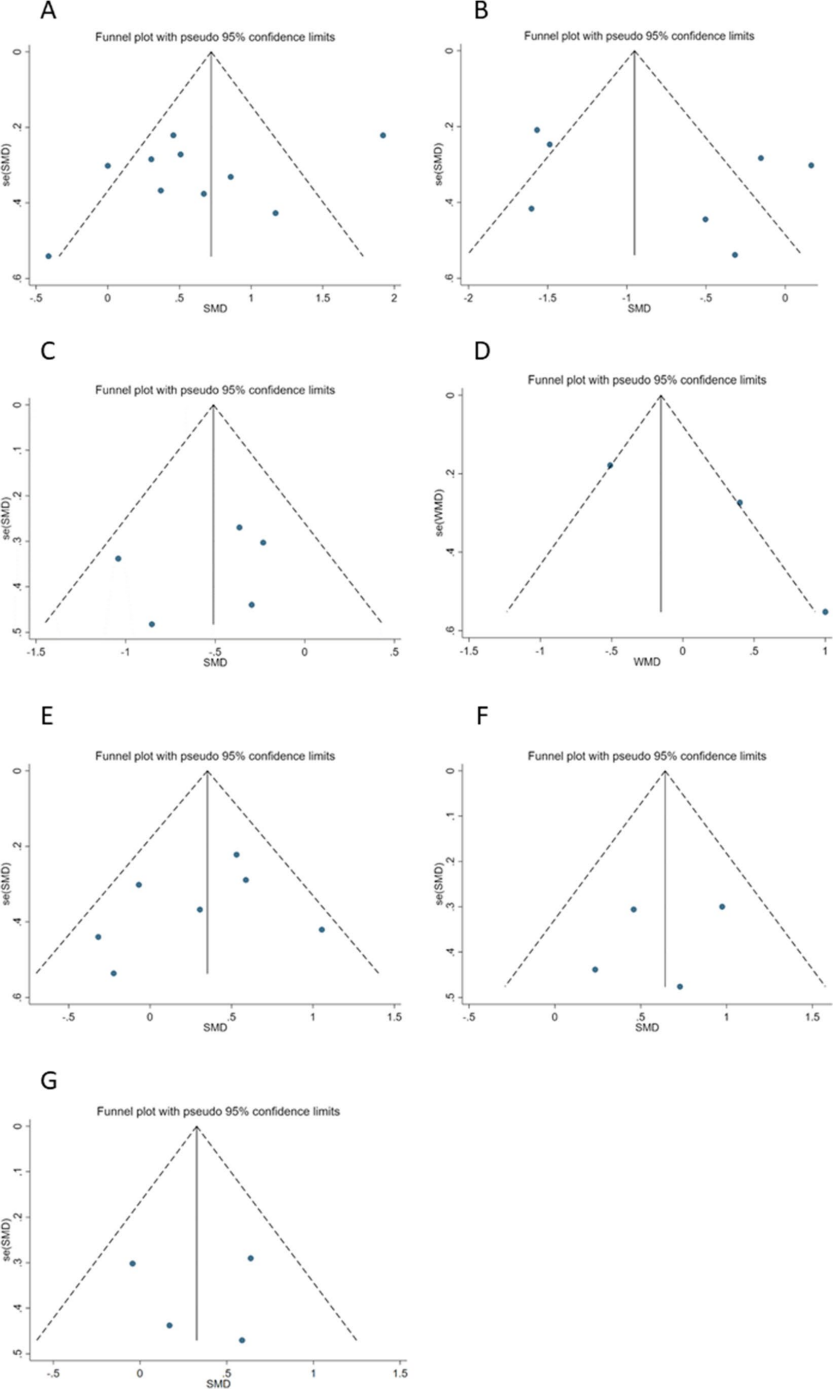
**A** Funnel plot for efficacy of upper extremity function. **B** Funnel plot for efficacy of pain intensity. **C** Funnel plot for efficacy of shoulder subluxation. **D** Funnel plot for efficacy of muscle spasticity. **E** Funnel plot for efficacy of general disability. **F** Funnel plot for efficacy of PROM of flexion. **G** Funnel plot for efficacy of PROM of abduction

Ten studies totaling 495 participants presented outcome for upper extremity function [21, 23, 32, 33, 35–40]. Meta-analysis revealed an association between the application of KT and upper extremity function (SMD 0.61, 95%CI 0.18 to 1.04) with significance heterogeneity across the studies (Fig. 3A, Fig. 4A). Exploratory sensitivity analysis revealed the study of Li et al. to be the likely source of this heterogeneity, with its exclusion from meta-analysis resulting in a revised *I*^2^ = 13% [42]. The possible causes may be more than 100 participants, while the sample size of other studies ranged from 14 to 84.

### Pain intensity

Pain intensity was reported in seven studies, totaling 364 participants [23, 32–34, 36, 38, 41]. Pooled data suggested significant difference in pain intensity with KT (SMD − 0.79, 95%CI − 1.39 to − 0.19, *I*^2^ = 85%) (Fig. 3B). The funnel plot (Fig. 4B) was slightly asymmetrical, and the Egger test did not indicate potential publication bias (*p* = 0.25).

A sensitivity analysis was conducted according to our protocol by excluding one study. Sensitivity analysis suggested that the pooled estimates or heterogeneity were not substantially modified by removing the included studies one by one.

### Shoulder subluxation

The pooled estimate based on 5 studies indicated significant effect of KT on alleviating shoulder subluxation (SMD − 0.50, 95%CI − 0.80 to − 0.20, *I*^2^ = 3%) without significant heterogeneity across the studies (Fig. 3C) [33, 34, 37, 40, 41]. The publication bias tests were not significant and the funnel plots (Fig. 4C) can be considered slightly asymmetrical and the Egger test did not indicate potential publication bias (*p* = 0.56).

### Muscle spasticity

Three studies (*n* = 52) reported muscle spasticity [21, 33, 39]. There was no statistically significant reduction in score of modified Ashworth scale (MAS), with KT (MD 0.19, 95%CI − 0.65 to 1.04) compared with conventional rehabilitation alone. There was significant heterogeneity across the studies (Fig. 3D). The results of publication bias showed the funnel plots was symmetrical and there was no significance of Egger’s regression (*p* = 0.29).

In the case of substantial heterogeneity, we explore possible causes by sensitivity analysis. When we remove the study of Yang et al. [39], the *I*^2^ statistics for heterogeneity reduced to 0%. The factor was identified as potential contributors to this observation was a modestly longer follow-up period compared with other studies.

### General disability

Data about the effects of KT on general disability were reported in 7 studies [23, 32–35, 38, 39]. Five trial evaluating general disability showed a favorable effect [32, 34, 35, 38, 39], and the remaining two trials reported no significance effects on general disability [23, 33]. We showed that KT have a positive influence on general disability (SMD 0.35, 95%CI − 0.10 to 0.59). No significant heterogeneity was found in the pooled outcomes, so a fixed-effect model was utilized in our study (Fig. 3E). The publication bias test was not significant with the Egger’s regression test (*p* = 0.41) and the funnel plot (Fig. 4E) was slightly asymmetrical.

### PROM of flexion

For PROM of flexion, the meta-analysis showed an improvement (SMD 0.63, 95%CI 0.28 to 0.98). A fixed-effect model was utilized in our study, since no significant heterogeneity was found in the pooled outcomes (Fig. 3F). The Egger regression test suggested there was no significance (*p* = 0.62) in agree with the result of funnel plot (Fig. 4F).

### PROM of abduction

No statistically significant difference in PROM of abduction was found with KT (SMD 0.32, 95%CI − 0.02 to 0.66) with no significant heterogeneity (Fig. 3G). The funnel plot (Fig. 4G) was slightly asymmetrical, and the Egger test did not indicate potential publication bias (*p* = 0.94).

### Adverse effects

Only 2 studies report whether there was the presence of adverse effects during the application of KT [34, 36]. Of those, only one study reported mild itching [36]. Overall, KT was well tolerated and only common and mild side effects were registered.

## Discussion

This is the first review to systematically summarize the benefits of KT on upper extremity function, including pain intensity, shoulder subluxation, general disability, and the PROM of flexion. In this study, we used original data from 12 randomized controlled trials of KT application to explore the effectiveness upper limb function in poststroke patients. Our study shows that the application of KT leads to a significant improvement upper extremity function, pain intensity, shoulder subluxation, general disability, and the PROM of flexion. However, there was no between-group difference in muscle spasticity or the PROM of abduction.

A recent overview that included 12 studies identified reduction in shoulder pain when combined with exercise in shoulder pain symptom patients [43],which is consistent with the present analyses. It has been proposed that the KT increases blood circulation and lymphatic drainage leading to a reduction in swelling and subsequently to relieve pain [44, 45]. The applied KT could also work by lifting the skin, resulting in a reduction of pressure on the subcutaneous nociceptors [46]. To date, the underlying pain-relief mechanism of KT remain confused [47]. Additionally, owing to the inconsistency in intensity, duration, and frequency of the KT application, we should take caution with our findings. For shoulder subluxation, we observed a moderate effect size, and heterogeneity was not obvious, even though different methods of measurement were utilized. Potential mechanisms of KT for shoulder subluxation seems plausible. Dr Kenso Kase suggested that KT can provide therapeutic effects, such as correction of joint misalignment and functional, proprioceptive stimulation [44, 48]. In terms of KT’s thickness and elasticity, it reduces swelling which could improve joint mobility, decrease intra-articular pressure [49]. Some reports have suggested there was significant difference between KT and conventional rehabilitation in muscle spasticity [17], while others suggested that there was no statistically significant improvement in muscle tone [18]. In our study, the pooled data shows that there was no significant in muscle spasticity. However, due to the limited number of included studies, the findings should be taken into consideration when analyzing the results thoroughly. The results of general disability and upper extremity function suggested that KT have positive impact on stroke patients. However, as function outcome, general disability and upper extremity function of stroke patients typically require long-term follow-up and large samples to detect effects on function [50]. Moreover, analyses of the flexion PROM and abduction PROM outcomes included small sample size and were likely not adequately power to detect an effect of KT. Overall, the observation that KT application improve upper extremity function and general disability compared with control groups, but does not reduce the muscle spasticity and the PROM of abduction, suggested that although KT does not improve the muscle tone and PROM, it does confer improve patients’ overall function.

There are four points deserved to think about. First, KT is a safe and effective method, while the mechanism of KT is not clear at present [51], its therapeutic effect may be by sticking KT into different directions and using different tensions [45]. However, the taping method seemed to vary across studies, including difference in target muscle, direction, tension, and concomitant treatment. And it could be found that the application was heterogeneous in terms of type of KT. The elasticity, perspiration, breathability, waterproofness, durability, adhesion, appearance, and other experiences are different in different type of KT. Therefore, our results should be interpreted with caution. Second, conventional rehabilitation or usual treatment may have differed across studies. The improvements in upper extremity function, pain intensity, shoulder subluxation, general disability, and the PROM of flexion demonstrated by studies are a combined effect of KT and conventional rehabilitation or usual treatment. It is unclear what extent contribution the KT made to the improvements because of varies of convention rehabilitation or usual treatment, including the type, intensity, and frequency. Additionally, the control group included a mixture of placebo-controlled and open-label studies, which should be taken into consideration when analyzing the results. Finally, there was a difference between included studies, including the follow-up time and sample size.

The main strength of this study is that we investigate a wide range of function outcomes, which adds exceptional value to this meta-analysis. As the population ages and stroke mortality declines [52], this is an area worthy of future research attention. More high-quality RCTs examining the effect of KT on upper limb function in patients with stroke are urgently needed. Furthermore, investigators should clearly and fully describe the details of the intervention, control, and outcomes. Our study has multiple limitations that need to be disclosed. Only 12 studies were included in this analysis, which limits the reliability of the results, and two trials were performed by the same group of authors. All studies included trials were single center. Additionally, meta-analyses are often limited to short-term effects, due to difficulties interpreting varied follow-up intervals and potential for other treatments during the follow-up phase [49]. However, moderation analyses are very useful in developing preventive strategies and designing appropriate interventions.

## Conclusion

In conclusion, the current systematic review and meta-analysis identified that KT could be used for improving upper limb function in stroke patients. However, due to the limited quality of the evidence currently available, the results should be treated with caution.

## Abbreviations

ADL: Activities of daily living
CI: Confidence intervals
CNKI: The China National Knowledge Infrastructure
Co. LTD: Limited company
KT: Kinesiology taping
MAS: Modified Ashworth scale
PRISMA: The preferred reporting items for systematic reviews and meta-analyses
PROM: Passive range of motion
PROSPERO: International prospective register of systematic reviews
PEDro: Physiotherapy evidence database
RCT: Randomized controlled trial
SMD: STD mean difference
